# Tackling the cancer burden: the economic impact of primary prevention policies

**DOI:** 10.1002/1878-0261.12812

**Published:** 2020-10-18

**Authors:** Jane Cheatley, Alexandra Aldea, Aliénor Lerouge, Marion Devaux, Sabine Vuik, Michele Cecchini

**Affiliations:** ^1^ Health Division Organization of Economic Cooperation and Development Paris France

**Keywords:** alcohol, cancer, healthcare expenditure, noncommunicable diseases, obesity, projection

## Abstract

Cancer is a noncommunicable disease (NCD) with increasing incidence and therefore constitutes a major public health issue. To reduce the health and economic burden of cancer, policy‐makers across the world have implemented a range of preventative interventions targeting risk factors with a known link to the disease. In this article, we examine the impact of six primary prevention interventions – related to physical inactivity, unhealthy diet or harmful alcohol use – on cancer‐related health outcomes and healthcare expenditure. Here, we used the OECD Strategic Public Health Planning for NCDs (SPHeP‐NCDs) model to quantify outcomes and costs for each intervention for years 2020–2050 across 37 countries. Results from the model indicate that all interventions could lead to a reduction in the number of new cancer cases, in particular those targeting harmful alcohol consumption. Introducing an alcohol tax, for instance, is estimated to reduce related cancer cases by 5619 a year or 174 193 by 2050. A breakdown of results by type of cancer revealed interventions had the largest impact on colorectal cancer with, on average, 41 140 cases avoided per intervention by 2050. In proportional terms, interventions had the greatest impact on new oesophageal and liver cancers. Findings from this article are designed to assist decision‐makers efficiently allocate limited resources to meet public health objectives.

## Graphical abstract

Cancer is a major public health issue. In response, policy‐makers have implemented a range of preventative interventions targeting key risk factors. Using OECD's Strategic Public Health Planning for Noncommunicable Diseases model, this article examines the health and economic impact of six primary prevention interventions targeting cancer. Findings can assist decision‐makers efficiently allocate resources to meet public health objectives.

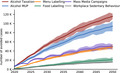

**Abbreviations**
ASIR: age‐standardized incident rate
BMI: body mass index
DALY: disability‐adjusted life year
MUP: minimum unit pricing
NCD: noncommunicable disease
OECD: Organization for Economic Cooperation and Development
PPP: purchasing power parity
SDG: Sustainable Development Goal
SPHeP‐NCD: Strategic Public Health Planning for NCDs
UN: United Nations
USD: United States Dollar
WHO: World Health Organization

## 1 Introduction

The impact of cancer on global health is well publicized. Using latest available data, the estimated number of cancer incident cases and deaths across genders and all groups in the world was approximately 18.1 million and 9.5 million, respectively [1]. At the population level, depending on a country's income status, this translates into an age‐standardized incident rate (ASIR) (a measure which takes into account differences in the age structure of populations being compared) between 110–342 and 113–278 per 100 000 people for men and women, respectively [2, 3]. For both genders, colorectal and lung cancers are two of the most common forms of cancer along with prostate cancer for men and breast cancer for women [4, 5].

Since 2007, the ASIR for cancer increased in 123 out of 195 countries analysed as part of the Global Burden of Disease study, particularly amongst high‐income countries due to ageing populations [6]. Conversely, over the same period, the cancer death rate declined in 145 countries [6].

In response to the growing number of people living with one or multiple NCDs, including cancer, global targets to reduce the NCD burden have been set. The United Nations (UN), as part of the Sustainable Development Goals (SDGs), which were adopted by Member States in 2015 in an effort to end poverty, protect the planet and improve the lives of all people, set a target of reducing premature mortality from NCDs by one‐third by 2030 [7]. The World Health Organization (WHO) in its *Action Plan for the Prevention and Control of NCDs* (2013–2020) proposed a consistent target [8].

One of the most effective, and cost‐effective [9], approaches to achieve these targets is to implement comprehensive policy packages addressing salient NCD risk factors – unhealthy diet, physical inactivity, harmful use of alcohol and tobacco use [8, 10]. Addressing key risk factors is essential for reducing cancer‐related burden given it is estimated that between 30% and 50% of all cancers are preventable [11]. For example, in the United Kingdom, Brown *et al*. [12] estimated that improvements in lifestyle behaviours can prevent 38% of cancers. This proportion is derived from an analysis which found approximately three in 20 cancer cases are caused by unhealthy diet and weight^i^, with this figure rising to three in 10 when adding tobacco use and exposure to UV radiation^ii^ [12].

Given the well‐established link between cancer and risk factors, decision‐makers have responded by implementing preventative interventions promoting healthy lifestyle behaviours [9]. Example interventions include taxes on unhealthy items, mass media public health campaigns and nutrition and warning labels for unhealthy foods and alcohol, respectively [13, 14]

Various projection studies have been undertaken to evaluate the health and economic impact of healthy lifestyle interventions. In 2010, Cecchini *et al*. [15] found policies targeting diet, physical activity and obesity (e.g. food advertising regulation and school‐based interventions) have the potential to reduce the incidence of lung, colorectal and female breast cancer. A systematic review by Kohler *et al*. [16] drew similar conclusions stating that high adherence to nutrition and physical activity guidelines is associated with a 10–61% reduction in overall cancer incidence. More recently, a 2019 modelling study found menu labelling; food labelling; mass media campaigns; workplace programmes targeting sedentary behaviours; prescription of physical activity; and public transport interventions were all effective in reducing the number of cancer cases [14]. Regarding harmful alcohol use, a study on the impact of alcohol prevention interventions concluded that excise taxes and brief interventions in primary care and the workplace were effective in reducing rates of alcohol‐related cancer: a 10% rise in alcohol prices, for instance, could cut alcohol‐related cancer rates by 2% [17].

The objective of this article is to develop the literature regarding the health and economic impact of interventions targeting risk factors linked to cancer using an advanced systems modelling tool. Based on a review of the literature, it is hypothesized the analysis will reveal significant health and economic gains for all interventions.

Findings from the article can assist decision‐makers efficiently allocate limited resources to interventions with the greatest level of impact, thereby helping them achieve ambitious NCD targets while containing costs.

The remainder of this article sets out the methodology used to quantify the impact of primary prevention interventions, followed by results from the microsimulation model used to undertake the analysis, and, finally, a discussion on the policy implications of key findings.

## 2 Materials and methods

### 2.1 OECD's Strategic Public Health Planning for NCDs model

OECD's Strategic Public Health Planning for NCDs (SPHeP‐NCDs) is a dynamic microsimulation model [18]. The model is used to quantify the impact of primary prevention policies on behavioural risk factors with a known link to cancer, namely unhealthy diets, physical inactivity and harmful alcohol consumption [19, 20, 21, 22]. In short, the model is designed to assess the impact of an intervention compared to ‘business‐as‐usual’ (i.e. counterfactual analysis in which no new intervention is introduced and provision of preventive and healthcare services remains at current levels specific to a country).

The microsimulation model consists of three core modules – a demographic, a risk factor and a disease module. The demographic module assigns each individual in the model a birth date, gender and migration status. This is designed to create synthetic life histories (i.e. from birth to death), which, when aggregated, reproduce population dynamics for a given country.

In the risk factor module, individuals are permanently allocated to a fixed quantile for each risk factor in the module, with a higher quantile representing a higher risk factor (e.g. a higher level of alcohol consumption). Based on the characteristics assigned within the demographic and risk factor modules, an individual has a certain risk (i.e. relative risk) of developing a disease, such as cancer, each year. Relative risks are based on those outlined in Global Burden of Disease study [23, 24].

Finally, the disease module simulates the disease pathway (incidence, fatality and remission) through events at the individual level. For cancers, incidence and mortality data are computed using the Institute for Health Metrics and Evaluation (IHME) data, which are broken down by age, gender and year [24]. Remissions are calibrated to complement the number of deaths against incident cases in a 5‐year timeline – that is individuals who do not die from cancer within 5 years of being diagnosed are considered to have recovered. In practice, for each incident case, the person is assigned a probability of dying of cancer within 5 years and, in the case of a predicted death, a time to death based on mortality data. Cancer deaths do not occur uniformly within the 5‐year timeline from diagnosis; instead, using data from IARC [25], each year is assigned a weight to reflect the fact that mortality is highest in the first year after diagnosis and declines thereafter. Additional information on the modelling of cancer diseases can be found elsewhere [14]. For this analysis, cancer cases directly related to the interventions are liver, breast, oesophageal and colorectal cancers, while lung cancer is considered unrelated (i.e. indirectly affected). Other cancers, such as mouth cancer, have not been included due to the lack of available cost data.

For each year, the model produces a cross‐sectional representation of the population, which is used to calculate health and economic outcomes associated with an intervention. The former (health outcomes) includes indicators such as life expectancy, disease prevalence and disability‐adjusted life years (DALYs) using disability weights. The latter (economic outcomes) calculates the healthcare costs of disease treatment based on a per‐case annual cost basis, which is extrapolated from national health‐related expenditure data. The additional cost of multimorbidity is also calculated and applied [14]. Detailed information on the OECD SPHeP‐NCD model is available online [18]: http://oecdpublichealthexplorer.org/ncd‐doc/.

Six public health interventions were included in the analysis. Interventions were selected for three reasons. First, each intervention has garnered significant discussion in countries under analysis and therefore of high interest to policy‐makers [14]. Second, interventions align with ‘Best Buy’ policies recommended by WHO, thereby providing further insight into policies previously deemed best practice [26]. Third, high‐quality evidence of each intervention's impact on the risk factor it targets was available in the academic literature. Interventions targeting tobacco were excluded given the depth of information currently available in the literature.

Modelling the impact of individual public health interventions requires four inputs: (a) a description of the target population, including age group and health status; (b) exposure of the target population to the intervention; (c) effectiveness of the intervention at the individual level; and (d) time to maximum effectiveness and effectiveness over time. Input values for each of the primary prevention interventions evaluated in this study are summarized in Table 1, which have been sourced from the academic literature.

**Table 1:** Input values for the model.

	Target age	Exposure (% eligible population)	Effectiveness
Menu labelling	> 5 years	12%	1.05–1.31% drop in BMI after 1 year
Food labelling	> 5 years	15%	0.40% lower BMI
Mass media campaigns	> 18 years	100%	60% increase in (at least) moderate activity after 1 month; 30% after 1 year; and 0% after 2 years
Workplace sedentary behaviour (SB)	18–65	2.31–6.95%	−72.78 min of SB per 8‐h workday
Alcohol tax (a price increase of 10%)	All	100%	Alcohol consumption: −4% to −7%
Minimum unit pricing	All	100%	Alcohol consumption: −0.6% to −3.3%

The analysis assumes interventions are implemented in 2019, with results expressed over the period 2020–2050. Thirty‐seven countries have been included in the analysis, which were chosen based on data availability (Table 2). Given the model uses a standardized approach, the analysis allows for cross‐country comparisons.

**Table 2:** Analysed countries.

Continent	Countries
Africa	South Africa
Asia	Japan
Australia	Australia
Europe	Austria, Belgium, Bulgaria, Croatia, Cyprus, Czech Republic, Denmark, Estonia, Finland, France, Germany, Greece, Hungary, Ireland, Iceland, Italy, Lithuania, Latvia, Malta, Netherlands, Norway, Poland, Portugal, Romania, Russian Federation, Slovak Republic, Slovenia, Spain, Sweden, Switzerland and United Kingdom
North America	Canada and Mexico

### 2.2 Primary prevention interventions

The six primary prevention interventions chosen for analysis, and their aligning risk factor, are summarized in this section.

#### 2.2.1 Menu labelling (risk factor targeted: unhealthy diet)

Models an intervention that legally requires all restaurants and food outlets to provide information on calorie content, as well as other nutrition information, such as sugar and salt, at the point‐of‐sale [27]. To assist customers interpret this information, nutrition content may be contextualized for example via percentage of daily intake; amount of physical activity required to expend calories in each item; and/or a traffic light system to visually represent overall nutritional content [27].

#### 2.2.2 Food labelling (unhealthy diet)

Models the impact of statutory measures requiring manufacturers or retailers to provide information on the nutritional composition of foods sold in supermarkets and other stores [28]. The intervention takes into account that not all calories consumed come from foods purchased in supermarkets and stores [29].

#### 2.2.3 Mass media campaigns (physical inactivity)

Models the impact of a health‐promoting mass media campaign encouraging people to lead a more active lifestyle. The campaign is delivered via traditional media channels, specifically, two 15‐s television paid commercials [30] combined with advertisements in printed media such as posters and leaflets as well as public relations events [31].

#### 2.2.4 Workplace programmes targeting sedentary behaviour (physical inactivity)

Models the impact of an employee‐sponsored programme designed to reduce sitting time through the use of sit–stand and treadmill desks. The intervention assumes sitting time is reduced by 72.78 min per workday (8 h) [32] and that 50% of eligible enterprises choose to participate [33].

#### 2.2.5 Alcohol tax (harmful alcohol use)

Models a 10% increase in the price of all alcoholic beverages due to an increase the tax rate. Given inputs for the model were based on studies estimating the impact of taxes on consumption, as opposed to sales, estimates for this intervention take into account consumption of alcohol from illicit sources. Price elasticities for alcohol were derived from a systematic review and meta‐analysis and estimated along three dimensions: type of beverage; age of drinkers; and category of drinking. This information was then combined with the level of alcohol consumption per capita in each country.

#### 2.2.6 Minimum unit pricing (harmful alcohol use)

Minimum unit pricing (MUP) sets a mandatory floor price per unit of alcohol or standard drink thereby targeting cheap alcohol beverages [34]. The intervention is modelled using the three dimensions: (a) the proportion of alcoholic beverages on the market that fall below a set minimum price threshold; (b) the average price increase, per unit of alcohol, for beverages in the low‐cost category; and (c) the impact the price increase has on alcohol consumption [35, 36].

## 3 Results

### 3.1 Impact on cancer‐related health outcomes

#### 3.1.1 Combined impact on all cancer types

Implementation of each intervention is expected to lead to a fall in the number of new cancer cases, in particular, interventions targeting the price of alcohol (Fig. S1). Specifically, an increase in the alcohol tax is estimated to reduce the number of new related cancer cases summed across all countries by 5619 per year (95% CI: 5597–5642) or, cumulatively, by 174 193 (95% CI: 173 425–174 962) for years 2020–2050. At the individual country level, alcohol taxation has the greatest impact in Austria, Czech Republic and Luxembourg with the proportion of new related cancer cases avoided for years 2020–2050 ranging between 0.51% and 0.55%. Introducing MUP is expected to reduce new related cancer cases per year by 4554 (95% CI: 4528–4580) [or 141 175 (95% CI: 140 473–141 877) by 2050]. For MUP, the Czech Republic is estimated to experience the biggest gain with 0.51% (95% CI: 0.36–0.66) of new cancer cases avoided followed by Austria [0.49% (95% CI: 0.40–59)] and Ireland [0.43% (95% CI: 0.31–0.55)].

Menu labelling and mass media campaigns are estimated to have the next largest impact resulting in 73 449 (95% CI: 73 227–73 670) and 70 271 (95% CI: 69 739–70 804) cancer cases avoided by 2050, respectively. For menu labelling, the proportion of new related cancer cases avoided is highest in Portugal, Mexico and South Africa (0.21–0.24%), while for mass media campaigns the Russian Federation, Hungary and Bulgaria stand to benefit most (0.35–0.44%). Workplace programmes targeting sedentary behaviour and food labelling are associated with the lowest impact with cancer cases avoided ranging from 23 823 (95% CI: 23 683–23 962) and 32 437 (95% CI: 32 180–32 695), respectively.

**Fig. 1:**
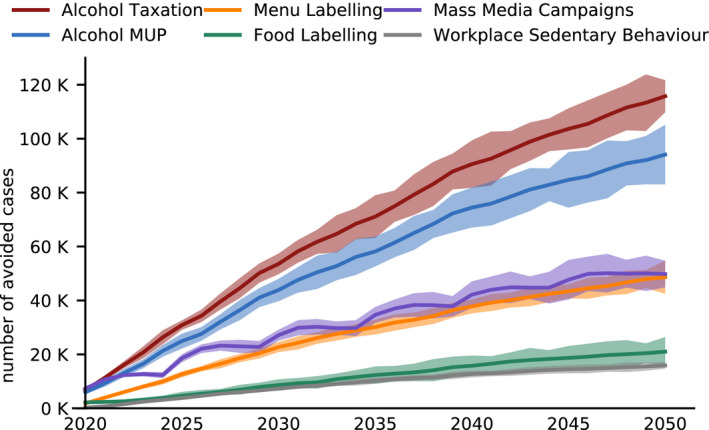
New cancer cases avoided over time by intervention (2020–2050). Shaded areas represent 95% CI. Source: OECD SPHeP‐NCD model, 2020.

An analysis of results over time indicates a large proportion of benefits associated with interventions take approximately 10 years to emerge (Fig. 1): on average across interventions, 47% of new cancer cases avoided occur in years 2020–2030 compared to 32% in 2031–2040 and 21% in 2041–2050.

#### 3.1.2 Impact by type of cancer

**Fig. 2:**
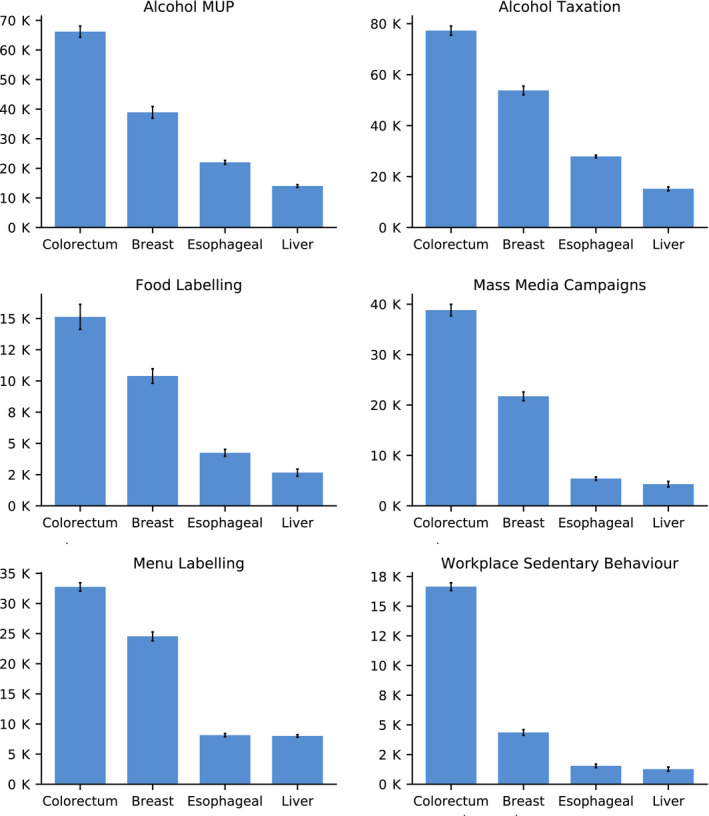
New cancer cases avoided by type of cancer and intervention by 2050. Vertical black lines represent 95% CI. Source: OECD SPHeP‐NCD model, 2020.

The interventions analysed had a direct impact on four types of cancers – breast, colorectal, oesophageal and liver cancer (Fig. 2). In gross terms, the average impact of each intervention was greatest for colorectal cancer [41 140 (95% CI: 40 070–42 211) cases avoided by 2050]. This was followed by breast cancer [25 627 (95% CI: 24 616–26 638)]; oesophageal cancer [11 547 (95% CI: 11 141–11 953)]; and liver cancer [7578 (95% CI: 7176–7980)].

In proportional terms, interventions targeting alcohol pricing are estimated to have the largest effect on oesophageal and liver cancers. For example, over years 2020–2050, MUP is expected to reduce, on average across countries, 0.71% (95% CI: 0.67–0.75) of new liver cancer cases with this figure increasing to 0.88% (95% CI: 0.86–0.90) for oesophageal cancers. Menu and food labelling, similarly, are most effective in reducing the number of new oesophageal and liver cancer cases, albeit to a lesser extent [e.g. 0.33% (95% CI: 0.32–0.34) of new oesophageal cancer cases for menu labelling]. Contrary to all other interventions, workplace interventions targeting sedentary behaviour has the greatest impact on new colorectal cancer cases [0.07% (95% CI: 0.06–0.07) over years 2020–2050].

#### 3.1.3 Impact by age and gender

**Fig. 3:**
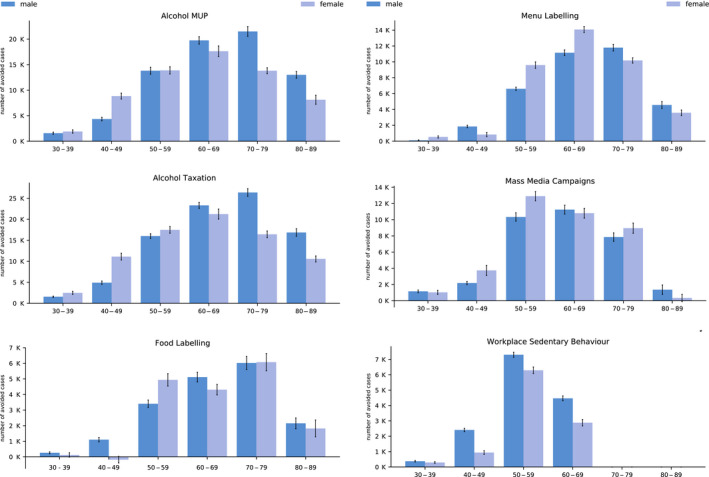
New cancer cases avoided by age, gender and intervention by 2050. Vertical black lines represent 95% CI. Source: OECD SPHeP‐NCD model, 2020.

The majority of new cancer cases avoided occur in those aged 50–79 years given this is the age where people commonly develop cancers under analysis and that interventions examined often target adults (Fig. 3). Across all countries and interventions for years 2020–2050, 29.46% of new cancer cases avoided are estimated to occur in those aged between 50 and 59 years, 29.61% for the 60–69 years age group and 22.89% for the 70–79 years age group. At the individual intervention level, the proportion of new cases avoided by age group are similar with the exception of workplace programmes targeting sedentary behaviour given it targets the working age population. Specifically, over half [57.09% or 13 598 (95% CI: 13 365–13 830) by 2050] of all new related cancer cases avoided are attributed to those aged 50–59 years compared to between 20% and 33% for the remaining interventions.

A breakdown of results by gender reveals new cancer cases avoided across all interventions are evenly split between men and women (Fig. 3). Women comprise a larger proportion of new related cancer cases avoided in regard to menu labelling [37 246 (95% CI: 36 488–38 004) vs. 36 203 (95% CI: 35 293–37 113) by 2050] and mass media campaigns [37 509 (95% CI: 36 048–38 970) vs. 32 763 (95% CI: 31 658–33 868)]. Conversely, men make up a larger proportion of cases for workplace programmes targeting sedentary behaviour, a higher alcohol tax, MUP and food labelling [e.g. for alcohol tax, 90 715 (95% CI: 89 160–92 270) vs. 83 478 (95% CI: 81 986–84 971) cases avoided by 2050].

### 3.2 Impact on cancer‐related health costs

**Fig. 4:**
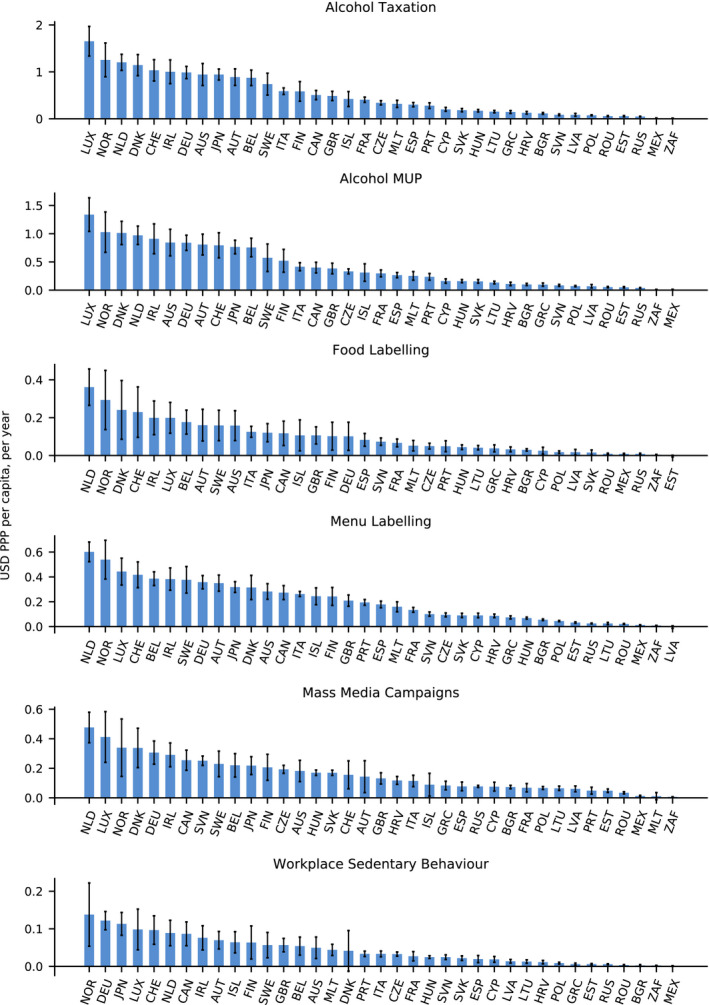
Impact on cancer‐related health expenditure by intervention and country, per capita, per year (USD PPP) (2020–2050). Vertical black lines represent 95% CI. Source: OECD SPHeP‐NCD model, 2020.

Each intervention results in health expenditure savings, albeit to differing extents (Fig. 4). A higher alcohol tax is associated with the greatest gain with each country, on average, saving USD (United States Dollars) 0.50 (95% CI: 0.49–0.50) per capita, per year in purchasing power parity (PPP) terms, which allows for accurate cross‐country comparisons. For MUP, this figure is expected to decrease to USD PPP 0.42 (95% CI: 0.41–0.42).

At the country level, the impact of alcohol pricing measures on health expenditure is estimated to be greatest in Luxembourg [per capita savings of USD PPP 1.34 (95% CI: 1.04–1.64) and USD PPP 1.65 (95% CI: 1.34–1.97) per year for MUP and higher alcohol tax, respectively] and Norway [USD PPP 1.03 (95% CI: 0.67–1.39) and USD PPP 1.26 (95% CI: 0.90–1.62), respectively]. Scaling‐up to the national level, for example, in Norway, translates into savings of USD PPP 236 million from a higher alcohol tax and USD PPP 193 million from MUP over years 2020–2050. Both Norway and Luxembourg are relatively small countries, in a larger country, such as Germany, savings from these two interventions increase to USD PPP 2.42 billion and USD 2.06 billion, respectively.

Interventions targeting unhealthy diets (i.e. food and menu labelling) save each country, on average, between USD PPP 0.10 (95% CI: 0.10–0.10) and 0.20 (95% CI: 0.20–0.21) per capita, pear year, respectively. Similarly, health expenditure savings on a per capita basis are greatest for countries such as the Norway and Luxembourg, as well as the Netherlands and Denmark. In the Netherlands, for instance, menu labelling is estimated to reduce health expenditure by USD PPP 328 million by 2050.

Finally, workplace programmes targeting sedentary behaviour and mass media campaigns can expect to reduce health expenditure, on average, by USD PPP 0.04 (95% CI: 0.04–0.05) 0.16 (95% CI: 0.15–0.16) per capita, per year, with countries such as Norway, the Netherlands, Germany and Japan benefiting most.

## 4 Discussion

Using OECD's SPHeP‐NCD advanced system modelling tool, this study estimates the health and economic impact of interventions targeting three preventable risk factors with a known link to cancer – unhealthy diets, physical inactivity and harmful alcohol consumption.

Modelling techniques used in this analysis stand out from existing modelling work for three key reasons. First, SPHeP‐NCD models the impact of interventions against real‐world counterfactual scenarios. As an example, in the absence of exposure to harmful alcohol consumption, the individual may still develop other diseases caused by different risk factors. Existing models under the same scenario typically assume the individual would live in good health for the rest of their life. Second, the model takes into account how costs change according to stage of disease (e.g. the extra cost of disease in the last year life for a patient with cancer). Finally, the model incorporates the additional costs of comorbidities, which is of greater policy relevance given interventions do not target single diseases.

Results from the model indicate each of the six interventions lead to a reduction in the number of new cancer cases. Interventions targeting the price of alcohol, namely through a higher tax and MUP, are estimated to yield the greatest health impact by reducing the number of new related cancer cases by 174 193 and 141 175 over years 2020–2050, respectively. This figure ranges between 23 822 and 73 448 for the remaining four interventions (menu labelling, food labelling, workplace programmes targeting sedentary behaviour and mass media campaigns). Results by type of cancer indicate that, under most interventions (i.e. all except menu labelling and workplace programmes targeting sedentary behaviour), the proportion of total oesophageal and liver cancers will experience the greatest decline. Finally, the majority of new cancer cases avoided occur in those aged 50–79 years, with the benefits split evenly between men and women.

Previous studies evaluating the impact of primary prevention interventions on cancer cases exist. For example, De Vries *et al*. [37] estimated that interventions targeting physical activity could reduce the number of new colon cancer cases by up to 5.1 per 100 000 people, per year in Europe, with this figure increasing to 11 for interventions targeting BMI. More recently, Webber *et al*. [38] estimated that a 5% reduction in BMI for the European population would lead to 185 cancer incidences avoided per 100 000 people. These findings align with those estimated in this article. Specifically, using the SPHeP‐NCD model, interventions targeting BMI (i.e. menu and food labelling) reduce cancer cases by between 0.3 and 3.1 per 100 000 people by 2050, depending on type of cancer (e.g. 3.1 colorectal cases per 100 000 for menu labelling). For interventions targeting physical activity (i.e. mass media campaigns and workplace sedentary programmes), this figure lies between 0.1 and 3.7 cases, with the greatest impact associated with mass media campaigns on colorectal cancer. Figures in this article are lower given it was assumed interventions affected a proportion of the population (e.g. between 12% and 15% for menu and food labelling) as opposed to the whole population and because changes in risk factors (e.g. in BMI) are lower compared to those evaluated by previous studies. Differences are also likely given the studies analysed different sets of countries.

As well as improved health outcomes, each intervention is expected to reduce health expenditure over the period 2020–2050 due to a fall in long‐term disease treatment and additional multimorbidity costs. The actual economic gains of the modelled interventions are likely to be higher given indirect benefits, namely from a boost in productivity (e.g. via reduced absenteeism), have not been included. These findings also correspond with previous analyses which conclude there is a robust economic case for implementing primary prevention policies targeting risk factors [39, 40, 41, 42].

Results from this study reaffirm the importance of addressing preventable risk factors to meet ambitious global NCD targets, such as the UN's goal to reduce premature mortality from NCDs by one‐third by 2030 (SDG 3.4). Analysis of progress towards achieving this target for most countries has been, to date, slow [43, 44]. Primary prevention is particularly pertinent for low‐ to middle‐income countries given these populations have lower levels of access to health services. Consequently, an avoided cancer case is more likely to translate into an avoidable death when compared to high‐income countries. Further, for all countries, reducing cancer cases frees up resources, which can be reallocated to treat nonpreventable diseases.

### 4.1 Limitations

Four noteworthy limitations are associated with this study. First, microsimulation models, such as the one used in this study, are a simplified version of the population they aim to model given they are heavily constrained by data availability. For example, cancer mortality data within the first 5 years of diagnosis were not available by age; therefore, the model assumes deaths from cancer within the first 5 years of diagnosis are constant across ages during this time. Second, the model does not take into account the interconnecting relationship between different risk factors due to a lack of robust available evidence as well as the effect interventions have on risk factors other than those they directly aim to modify. As an example, this study analysed the impact of interventions boosting physical activity on cancer cases directly; however, it did not include how an increase in physical activity may reduce air pollution and thus future cases of lung cancer. Third, the model assumes the impact of interventions on risk factors leading to cancer cases disappears soon after exposure given the paucity of long‐term impact data. Lastly, estimated costs exclude the impact of interventions on the labour market such as change in absenteeism, presenteeism and premature mortality. Due to limitations two, three and four, it is likely the model underestimates the impact of interventions on total cancer cases and their associated costs.

## 5 Conclusion

Findings from this study reveal interventions targeting unhealthy diets, physical inactivity and harmful alcohol consumption lead to an increase in the number of new cancer cases avoided. Consequently, demand for disease treatment will fall leading to a reduction in costs and an improvement in the financial sustainability of the health system. These results highlight the health and economic benefits associated with primary prevention interventions targeting cancer.

## Conflict of interest

The authors declare no conflict of interest.

## Author contributions

JC was responsible for writing the article. MC supervised the work and codrafted the article. AL and AA were the programmers. MD contributed to the methodology, sourcing of data and economic evaluation of interventions. SV contributed to the methodology, sourcing of data and economic evaluation of interventions.

## Supporting information


**Fig. S1.** New cancer cases avoided by country and intervention by year 2050.

## Data Availability

Data inputs to the SPHeP‐NCD model were obtained from publically accessible databases such as the Institute for Health Metrics and Evaluation and the Global Cancer Observatory (International Agency for Research on Cancer). Inputs to model the effectiveness of different interventions were obtained from the academic literature including meta‐analyses and systematic reviews (including those undertaken by the OECD, which were published in peer‐reviewed journals). Health expenditure data was obtained from country‐specific databases, which require potential users to apply for access. The methodology used to undertake the analysis is described in this article with further details provided on OECD's Public Health website (here: http://oecdpublichealthexplorer.org/ncd‐doc/). Finally, outputs from model are made publically available on OECD's Public Health website, which allows users to download results as Excel files.

## Footnotes

^i^ Unhealthy weight and diet: This includes being overweight or obese, drinking alcohol, eating too little fibre, eating processed meat and doing too little physical activity [12].

^ii^ Unhealthy living: This includes smoking, being overweight or obese, being overexposed to UV radiation, drinking alcohol, eating too little fibre, eating processed meat and doing too little physical activity [12].
