# Supplementary material for: Tackling the cancer burden: the economic impact of primary prevention policies
Source: Mol Oncol. 2020 Oct 18;15(3):779–89. doi: 10.1002/1878-0261.12812 (PMC7931126; doi:10.1002/1878-0261.12812)
Supplement: Supplementary file 1 — Fig. S1. New cancer cases avoided by country and intervention by year 2050. [file MOL2-15-779-s001.pdf]

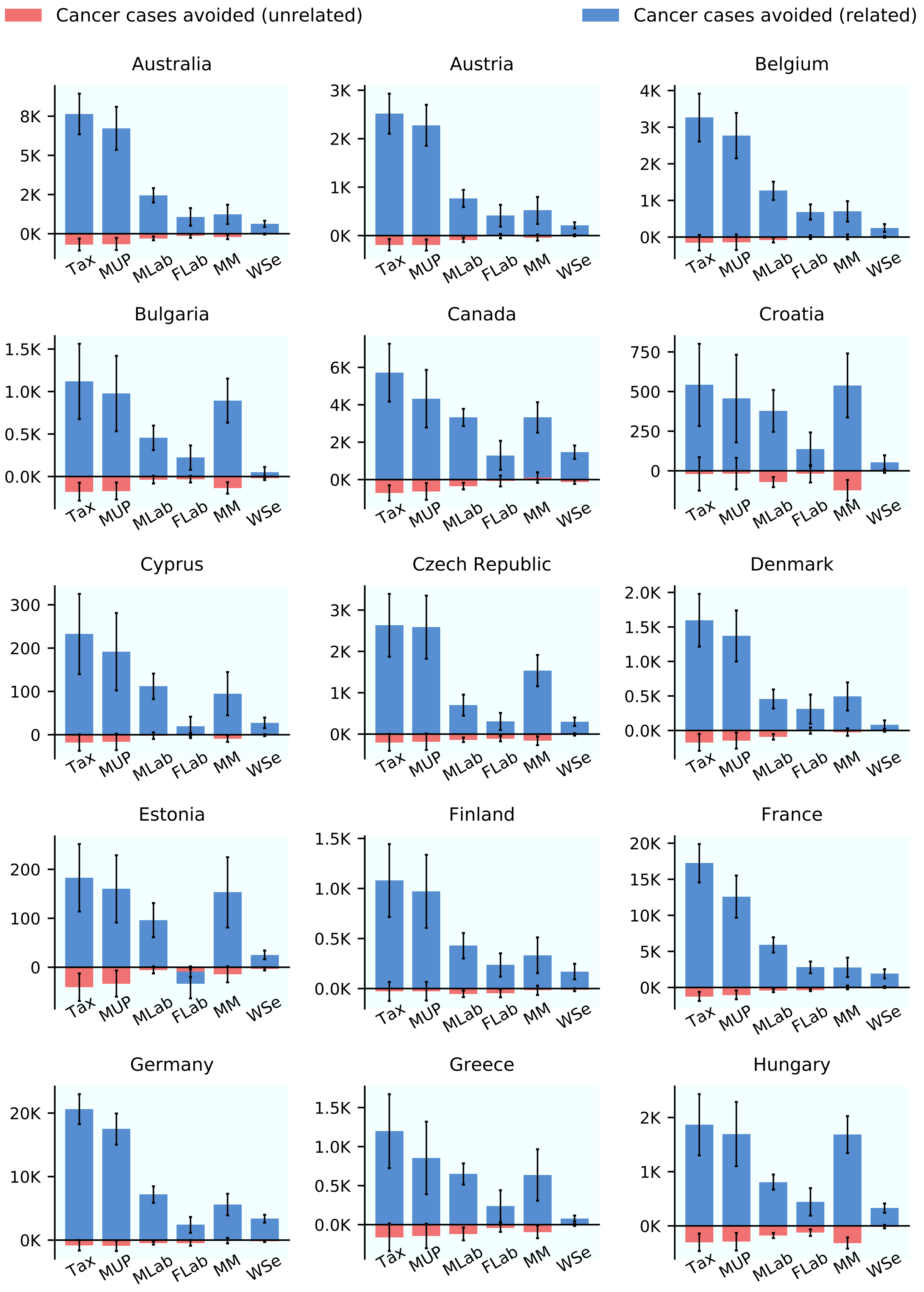
Cancer cases avoided (unrelated)
Cancer cases avoided (related)
Australia
Austria
Belgium
Bulgaria
Canada
Croatia
Cyprus
Czech Republic
Denmark
Estonia
Finland
France
Germany
Greece
Hungary
Tax
MUP
MLab
FLab
MM
WSe

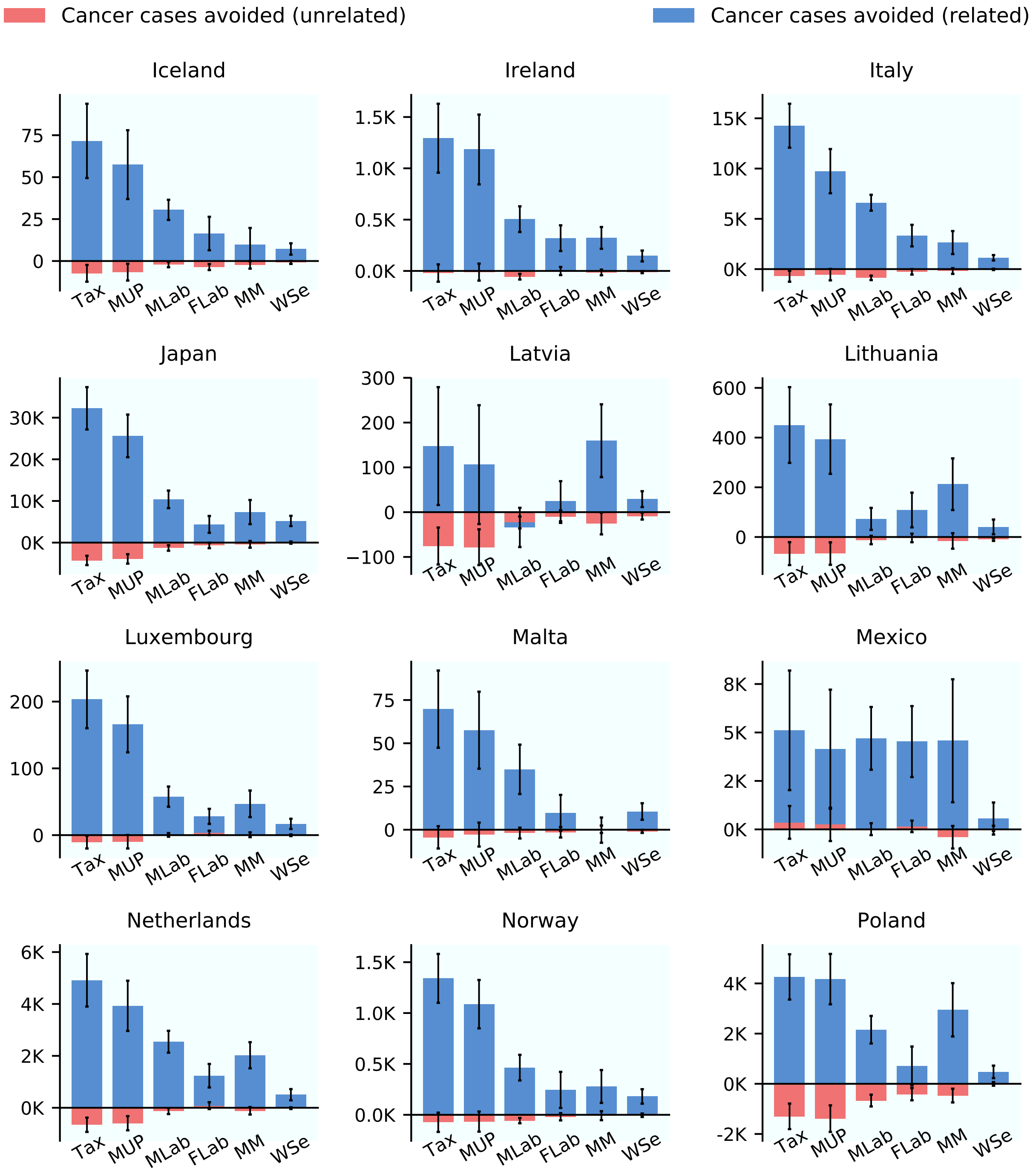

Cancer cases avoided (unrelated)
Cancer cases avoided (related)
Iceland
Ireland
Italy
Japan
Latvia
Lithuania
Luxembourg
Malta
Mexico
Netherlands
Norway
Poland
Tax
MUP
MLab
FLab
MM
WSe

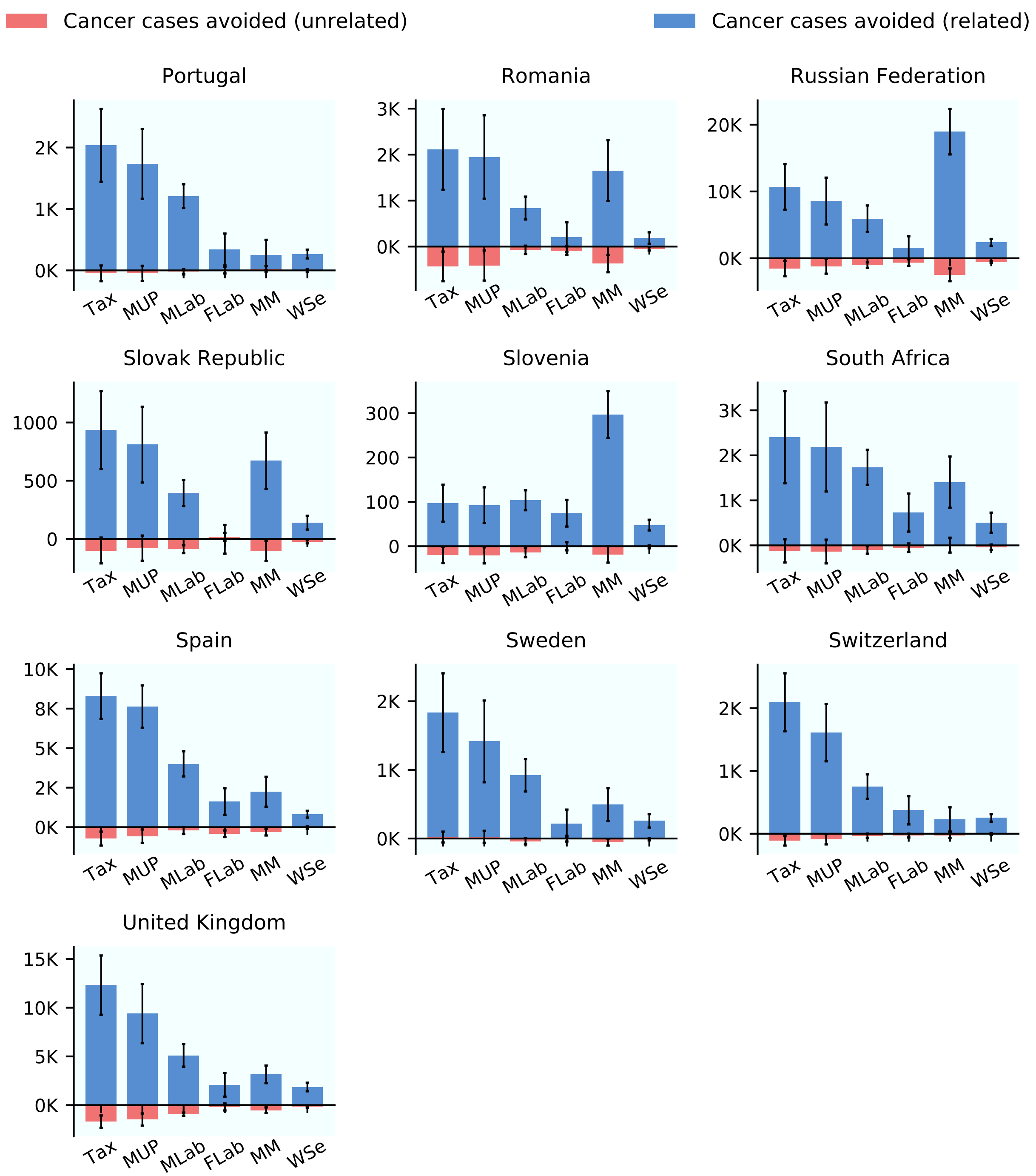

Figure S1 - New cancer cases avoided by country and intervention by year 2050

Note: The y-axis represents the total number of cancer cases avoided combined for years 2020-2050 as a result of each intervention, by country. Cancers cases avoided related to the intervention refer to liver, breast, esophageal and colorectal cancer cases. Cancer cases unrelated to the intervention refer to lung cancer cases. Cancer cases avoided which are negative (i.e. unrelated cancer cases) represent additional cancer cases caused by the intervention, while positive cancer cases avoided (i.e. related cancer cases) represent a decline in cancer cases.

Tax-Alcohol Taxation, MUP-Alcohol Minimum Unit Pricing, MLab-Menu Labelling, FLab-Food Labelling, MM-Mass Media Campaigns, WSe-Workplace Sedentary Behaviour, vertical black lines represent 95% CI

Source: OECD SPHeP-NCD model, 2020
